# Acquisition of Growth-Inhibitory Antibodies against Blood-Stage *Plasmodium falciparum*

**DOI:** 10.1371/journal.pone.0003571

**Published:** 2008-10-29

**Authors:** Fiona J. McCallum, Kristina E. M. Persson, Cleopatra K. Mugyenyi, Freya J. I. Fowkes, Julie A. Simpson, Jack S. Richards, Thomas N. Williams, Kevin Marsh, James G. Beeson

**Affiliations:** 1 The Walter and Eliza Hall Institute of Medical Research, Parkville, Victoria, Australia; 2 Department of Medical Biology, University of Melbourne, Parkville, Victoria, Australia; 3 Kenya Medical Research Institute, CGMRC/Wellcome Trust Collaborative Program, Kilifi, Kenya; 4 Centre for Molecular, Environmental, Genetic and Analytic Epidemiology, University of Melbourne, Carlton, Victoria, Australia; London School of Hygiene & Tropical Medicine, United Kingdom

## Abstract

**Background:**

Antibodies that inhibit the growth of blood-stage *Plasmodium falciparum* may play an important role in acquired and vaccine-induced immunity in humans. However, the acquisition and activity of these antibodies is not well understood.

**Methods:**

We tested dialysed serum and purified immunoglobulins from Kenyan children and adults for inhibition of *P. falciparum* blood-stage growth *in vitro* using different parasite lines. Serum antibodies were measured by ELISA to blood-stage parasite antigens, extracted from *P. falciparum* schizonts, and to recombinant merozoite surface protein 1 (42 kDa C-terminal fragment, MSP1-42).

**Results:**

Antibodies to blood-stage antigens present in schizont protein extract and to recombinant MSP1-42 significantly increased with age and were highly correlated. In contrast, growth-inhibitory activity was not strongly associated with age and tended to decline marginally with increasing age and exposure, with young children demonstrating the highest inhibitory activity. Comparison of growth-inhibitory activity among samples collected from the same population at different time points suggested that malaria transmission intensity influenced the level of growth-inhibitory antibodies. Antibodies to recombinant MSP1-42 were not associated with growth inhibition and high immunoglobulin G levels were poorly predictive of inhibitory activity. The level of inhibitory activity against different isolates varied.

**Conclusions:**

Children can acquire growth-inhibitory antibodies at a young age, but once they are acquired they do not appear to be boosted by on-going exposure. Inhibitory antibodies may play a role in protection from early childhood malaria.

## Introduction

*Plasmodium falciparum* malaria is a leading cause of childhood mortality, with around 1 million deaths annually [1]. In malaria-endemic areas effective immunity against malaria develops after repeated exposure that limits blood-stage parasitemia and prevents symptomatic illness and severe complications [2]. Antibodies that inhibit blood stage replication of *P. falciparum* are believed to be important in mediating both acquired immunity and immunity generated by candidate blood-stage vaccines [3], [4], [5]. Serum antibodies that inhibit parasite growth *in vitro* have been isolated from clinically immune individuals, but are rarely detected in malaria-naïve individuals [3], [5]. Early studies suggest that these “growth-inhibitory” antibodies are acquired in an isolate-specific manner following convalescence from acute infection [6]. However, the role of growth-inhibitory antibodies in protection from clinical disease in humans, or their function *in vivo*, has not been established. Furthermore, there is limited knowledge of the acquisition of these antibodies and it is unclear whether inhibitory antibodies can be acquired quickly after limited exposure or instead require repeated exposure over an extended period.

Inhibitory antibodies are thought to mainly act by inhibiting erythrocyte invasion through targeting merozoite surface antigens and invasion ligands [7], [8]. Data from animal studies support an important role for antibodies against merozoite antigens in immunity [9], [10]. Merozoite surface protein 1 (MSP1), apical membrane antigen 1 (AMA1), erythrocyte binding antigens (EBAs) and *P. falciparum* reticulocyte-binding homologues (PfRh proteins) are implicated as important targets of acquired human inhibitory antibodies [11], [12], [13], [14], and polyclonal and monoclonal antibodies produced in experimental animals against these antigens can inhibit erythrocyte invasion *in vitro* [7], [10], [12], [15], [16], [17]. Studies have found variable associations between the level of antibodies to recombinant merozoite antigens and protective immunity [18], [19], [20], [21], [22], [23], [24], [25], [26], [27]. However, measuring antibodies to recombinant merozoite proteins has significant limitations and antibody reactivity in these assays may not reflect functional activity. Epitope specificity and the affinity of antibodies are likely to be essential for inhibitory activity, and are not measured by standard ELISA. Furthermore, antibodies that can block the inhibitory activity of other antibodies have been described [28], [29], and antibodies may also act by inhibiting the processing of antigens required for erythrocyte invasion [29], [30]. These findings further emphasize the need for a greater understanding of growth-inhibitory antibodies, which is likely to be essential for the development and testing of blood-stage vaccines.

In order to understand the acquisition of human inhibitory antibodies and their potential role in immunity, we have evaluated the presence of inhibitory antibodies, and their association with active infection and markers of blood-stage malaria exposure and immunity, across different age groups and between populations with different transmission levels. Furthermore, we evaluated the relationship between inhibitory antibodies and antibodies to recombinant MSP1-42, which is a leading vaccine candidate that has been shown to induce growth-inhibitory antibodies [31], [32], [33], and the C-terminal fragment of which (MSP1-19) has been identified as a target of human inhibitory antibodies [11], [14].

## Methods

### Study site and population

The main study site of Ngerenya, Kilifi district, Kenya has been described previously [34], [35]. Ngerenya experiences seasonal malarial transmission following the “long” rains in May–July and the “short” rains in November, and in 1997/1998 the predicted entomologic innoculation rate (EIR) was 10 infective bites/person/year. Serum samples collected cross-sectionally from two separate Ngerenya cohorts were used. The first set (n = 150) were selected randomly from samples collected in September 1998 from Ngerenya adults and children (n = 354), as part of a longitudinal cohort study involving randomly selected households [34]. After baseline serum sample collection, individuals were followed-up for 12 months by active surveillance for symptomatic illness [34]. Study participants of the Ngerenya 1998 cohort were divided into 3 age groups, in concordance with age-associated incidence of clinical malaria in the area [34]; 2–5 years, n = 43; 6–14 years, n = 57 and 18–81 year olds, n = 50. The second set of samples was collected from the same village in October 2002 and comprised 237 children aged 1–8 years (1–5 years, n = 129; 6–8 years, n = 108). This subset was derived from a larger cohort of children (<8 years, n = 297) from randomly selected Ngerenya households [36]. We included all serum samples that were available from children ≥1 year of age.

Written informed consent was obtained from all participants, or their guardians, in the study. Ethical approval was obtained from the Ethics Committee of the Kenya Medical Research Institute, Nairobi, Kenya and from the Human Research Ethics Committee, the Walter and Eliza Hall Institute, Melbourne, Australia.

### Growth Inhibition Assays (GIAs)

The *P. falciparum* lines 3D7 and W2mef were cultured *in vitro* [37] and synchronised by alternate day re-suspension in 5% D-sorbitol (Sigma, St Louis, MO, USA) in water. Growth assays were performed over two cycles of parasite replication and parasitemia was measured using flow cytometry (FacsCalibur, Becton, Dickinson, Franklin Lakes, NJ) [13], [38]. Assays were set-up with mature trophozoites and schizonts, at 1% haematocrit and 0.3% starting parasitemia (50 µl/well) in 96 well U-bottom culture plates (Becton Dickinson, Franklin Lakes, USA) with pre-treated serum (5 µl) from cohort members or from non-exposed United Kingdom or Australian donors (n = 7 to 10). On all plates, PBS was included as a non-inhibitory control and 1F9 anti-AMA1 monoclonal antibody (0.5 mg/ml) [39] was included as an inhibitory control. Parasite growth for each sample is expressed relative to plate PBS controls. All samples were tested in duplicate in two separate assays.

To remove potential growth inhibitors or enhancers, serum samples were dialysed against PBS using 50 kDa MWCO microdialysis tubes (2051, Chemicon, Temecula, CA, USA) and subsequently reconstituted to original volume using centrifugal concentration tubes (100 kDa MWCO; Pall Corp, Ann Arbor, MI, USA) prior to testing in GIAs [38]. Purification of immunoglobulins (Ig) was performed by ammonium sulphate precipitation, as described [38].

### Enzyme-Linked Immunosorbent Assays (ELISAs)

Serum IgG recognising components of schizont protein extract and recombinant MSP1-42 was measured by ELISA [13]. Schizont protein extract was prepared from 3D7 cultures using standard methods. Recombinant MSP1-42 and MSP1-19 (3D7) proteins were kind gifts from Carole Long (NIH, Bethesda) and Paul Gilson (WEHI, Melbourne), respectively. Samples were tested in duplicate together with serum from 7–10 non-exposed United Kingdom or Melbourne donors. Positive and negative controls were included to allow standardisation.

### Statistical analysis

Statistical analysis was performed using Stata Version 9 (StataCorp, College Station, Texas, USA). The association between continuous variables was assessed using Students t-test or one-way ANOVA, or by Wilcoxon rank-sum test or Kruskal-Wallis test where appropriate. The association between categorical variables was assessed using a chi-square test. Pearson and Spearman's rank correlation coefficients were used to assess the association between two continuous variables. For the Ngerenya 1998 samples, the male to female ratio varied across the three age groups so the association between gender and the variables of interest was assessed separately within each age group. Data exclusions were due to insufficient sample for GIAs (Ngerenya 1998; 3D7, n = 7 and W2mef, n = 8), and outliers (Ngerenya 1998; 3D7, n = 3 and W2mef, n = 4). Survival analysis was performed using the Cox proportional hazards method. Malaria was defined as symptoms of fever or observed fever together with a parasitemia of >2500 parasites/µl.

#### Definitions

In the Ngerenya 1998 cohort, individuals with antibody levels equal to or greater than the 75^th^ percentile value for ELISA data were defined as ‘*high ELISA responders*’. Individuals whose sera inhibited growth below the approximate median value for each parasite line (3D7, <45% growth [% of control], n = 66; W2mef, <35% growth, n = 69) were defined as ‘*high inhibitors*’. To examine breadth and strength of responses, individuals were defined as ‘*strong inhibitors*’ (highly inhibitory of growth of both parasite lines (i.e. 3D7 <45% growth plus W2mef <35% growth)), and ‘*weak inhibitors*’ as those samples that were not strongly inhibitory of either 3D7 or W2mef (3D7 ≥45% growth plus W2mef ≥35% growth). The remaining samples were termed ‘*intermediate inhibitors*’.

## Results

### Acquisition of antibody to blood-stage antigens and MSP1-42 increases with age

**Figure 1:**
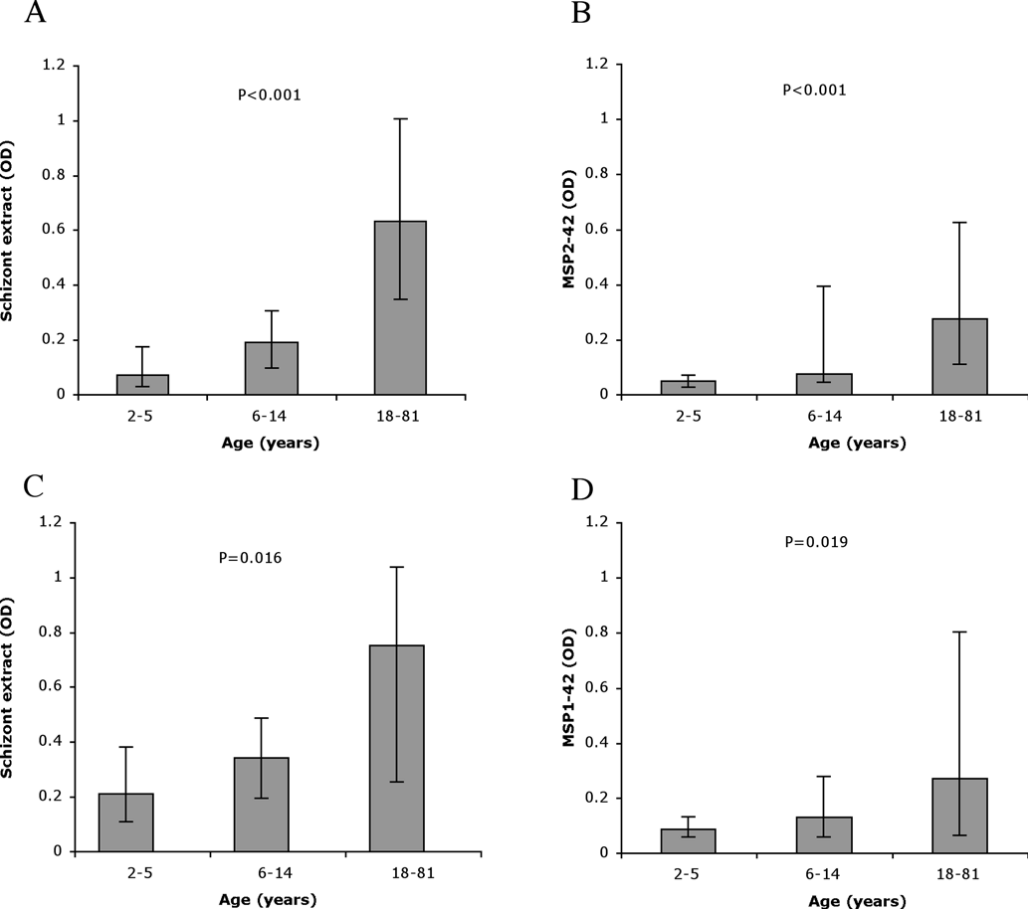
Levels of IgG to schizont protein extract and recombinant MSP1-42, according to age and parasitemic status, among samples from the Ngerenya 1998 cohort. A, B: Median absorbance (Optical Density, OD) to schizont protein extract and MSP1-42, respectively, for aparasitemic participants (n = 91). C, D: Median absorbance to schizont protein extract and MSP1-42, respectively, for parasitemic participants (n = 59). Sera were used at a dilution of 1∶1000. Error bars represent the inter-quartile range. P values were calculated using a Kruskal-Wallis test. All samples were tested in duplicate. The difference in median IgG levels for aparasitemic compared to parasitemic participants was not significant for schizont extract or MSP1-42 (P = 0.082 and P = 0.14, respectively).

Among the 150 individuals selected from the Ngerenya 1998 cohort, there was an age-related decrease in the prevalence and density of *P. falciparum* infection, reflecting the acquisition of immunity in the population (Table 1) [34]. Samples from this cohort were tested for IgG to schizont protein extract (a marker of exposure to blood-stage malaria and acquired immunity) and recombinant MSP1-42 (a major merozoite antigen and vaccine candidate) by ELISA. Levels of IgG to schizont extract and MSP1-42 were significantly correlated (Spearman's rho, r_s_ = 0.79, P<0.001). Median IgG levels to schizont extract and MSP1-42 increased with age in both aparasitemic and parasitemic individuals (P<0.02) (Figure 1 A–D). In concordance, the proportion of high responders to both antigens increased significantly with age (Table 1). There was no significant association between parasitemic status or gender and IgG levels against schizont extract or MSP1-42.

**Table 1:** ELISA and growth inhibition data for Ngerenya 1998 cohort.

Characteristics				Parasitemic status			Age group ( years)			
			n (%)	Aparasitemic n(%)	Parasitemic n(%)	P-value*	2–5 (n = 43)	5–14 (n = 57)	18–81 (n = 50)	P-value*
*P. falciparum* infection		Positive	59 (39.3)	-	59 (100)		13 (30.2)	34 (59.7)	12 (24.0)	0.001
		Density^a^/ul	0 (0–365)	-	682 (225–1827)		3712 (1151–12354)	762 (225–1452)	255 (180–380)	0.002
ELISA	MSP1 42	High Responders (%)^b^	37 (24.6)	22 (24.1)	15 (25.4)	0.862	2 (4.7)	11 (19.3)	24 (48.0)	<0.001
	Schizont extract	High Responders (%)^b^	37 (24.7)	25 (27.4)	12 (20.3)	0.322	1 (2.3)	8 (14.0)	28 (56.0)	<0.001
GIA	3D7	High Inhibitors (%)^c^	66 (47.1)	39 (45.9)	27 (49.1)	0.710	22 (56.4)	25 (47.2)	19 (39.6)	0.294
	W2mef	High Inhibitors (%)^d^	69 (50.0)	42 (49.4)	27 (50.9)	0.861	26 (70.3)	27 (49.1)	16 (34.8)	0.006
	3D7/W2mef	Responders (%)^e^								
		Strong	45 (33.1)				15 (40.5)	17 (32.1)	13 (23.3)	0.009
		Intermediate	40 (29.4)				16 (43.2)	16 (30.2)	8 (17.4)	
		Weak	51 (37.5)				6 (16.2)	20 (37.7)	25 (54.4)	

NOTES:

* P values calculated using a Chi-squared test or Kruskal-Wallis test.

a Median (25th–75th percentiles).

b High ELISA responders are those with absorbance≥the 75th percentile.

c High inhibitors to 3D7 are those with parasite growth less than the approximate median (i.e. <45% of control).

d High inhibitors to W2mef are those with parasite growth less than the approximate median (i.e. <35% of control).

e Strong responders are those with high GIA response to both 3D7 and W2mef. Weak responders are those with low GIA response to both 3D7 and W2mef. Intermediate responders are those remaining.

Abbreviations: ELISA, Enzyme-Linked ImmunoSorbent Assay; MSP1 42, Merozoite Surface Protein1 ; GIA, Growth Inhibition Assay. 3D7 and W2mef represent two different parasite lines.

### Age and exposure-related pattern of acquisition of growth-inhibitory antibodies

**Figure 2:**
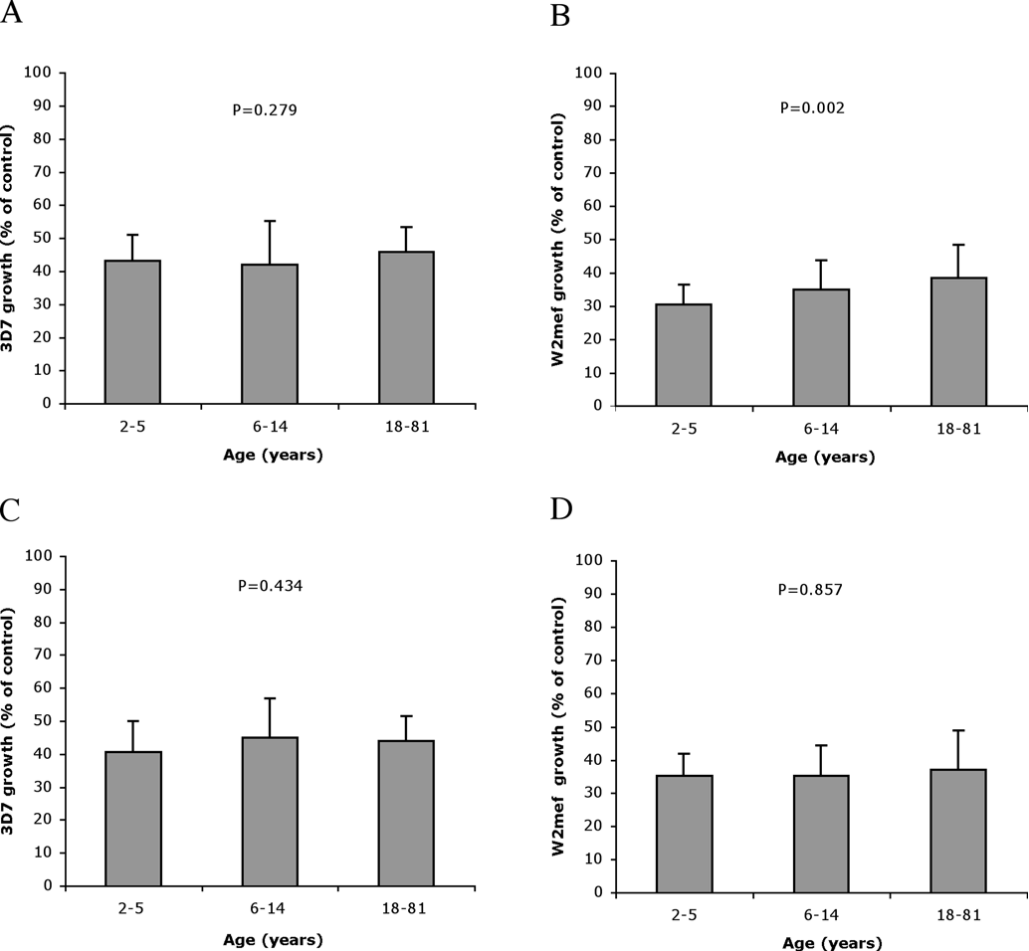
*In-vitro* growth inhibition of parasite lines 3D7 and W2mef, according to parasitemic status, among samples from the Ngerenya 1998 cohort. A, B: Mean growth of 3D7 and W2mef, respectively, for aparasitemic participants. C, D: Mean growth of 3D7 and W2mef, respectively, for parasitemic participants. Growth is expressed relative to control (PBS). Error bars represent standard deviation. P values were calculated using a one-way ANOVA. All samples were tested in duplicate in two separate assays. For all samples, including parasitemic and aparasitemic individuals together, mean growth (%, ±SD) was: W2mef (n = 138) 31.8±6.5, 2–5 years; 35.1±9.0, 6–14 years; 38.1±10.3, 18–81 years; 3D7 (n = 140) 42.4±8.3, 2–5 years; 44.2±12.4, 6–14 years; 45.4±7.5, 18–81 years.

In contrast to the ELISA data, we did not observe a marked increase in growth-inhibitory antibodies with increasing age among the Ngerenya 1998 samples (Figure 2). Age associated profiles of inhibition differed slightly for the two parasite lines despite a significant positive correlation between growth of 3D7 and W2mef (r = 0.62, P<0.001). A small, statistically significant reduction in the level of parasite growth inhibition with increased age was observed for W2mef (P = 0.007), but not for 3D7 (P = 0.365). After stratifying by parasitemic status, a significant association between reduced growth inhibition of W2mef with age was seen for aparasitemic participants only (P = 0.002, Figure 2B). The proportion of highly inhibitory samples was greatest in young children and decreased with age (3D7, P = 0.294 and W2mef, P = 0.006) (Table 1).

**Figure 3:**
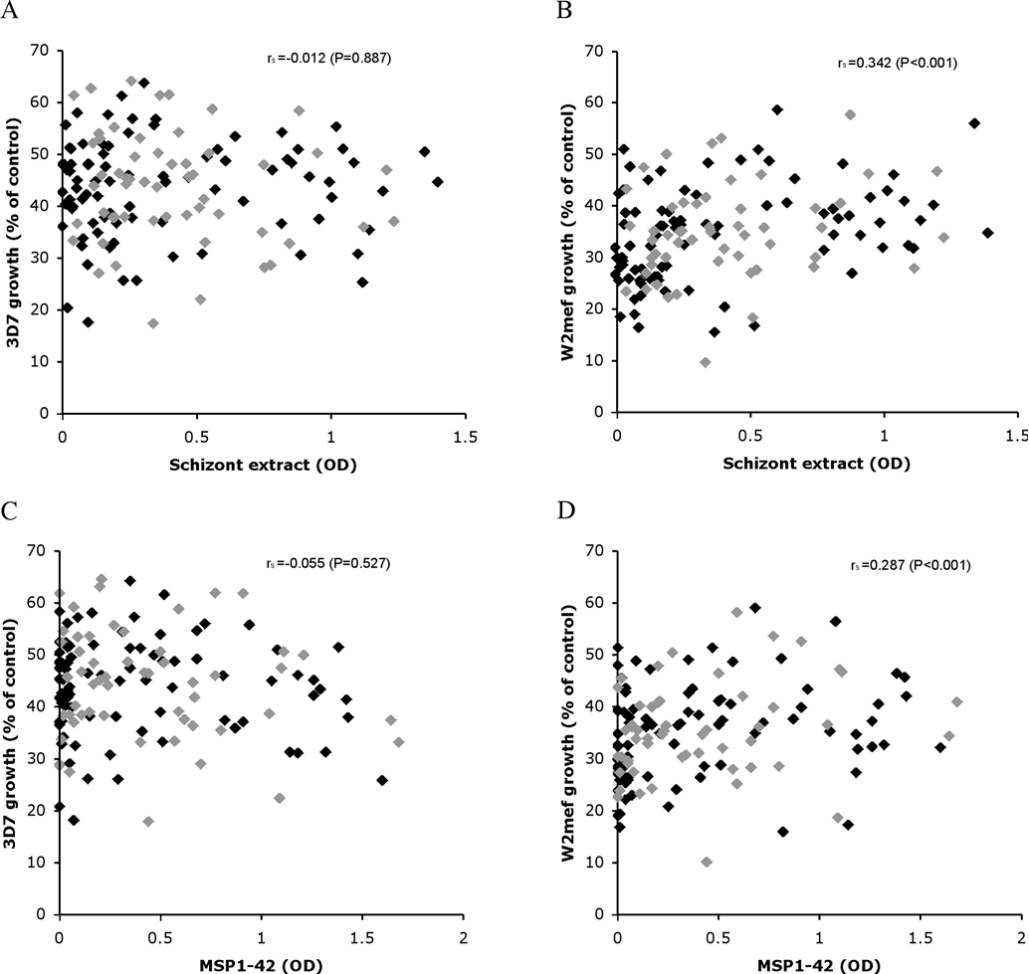
The association between *in vitro* growth of parasite lines 3D7 or W2mef and IgG measured by ELISA, according to parasitemic status, for the Ngerenya 1998 cohort. A, B: Correlation between ELISA response to schizont extract and 3D7 or W2mef growth, respectively. C, D: Correlation between ELISA response to MSP1-42 and 3D7 or W2mef growth, respectively. r_s_ values represent Spearman's rank correlation coefficients. Results for aparasitemic and parasitemic individuals are represented by black or grey diamonds, respectively. Samples used in growth-inhibition assays were dialysed serum.

Examining associations between growth inhibitory antibodies and exposure, a weak positive correlation was seen between greater W2mef growth (i.e. reduced growth inhibition) and IgG to schizont extract (r_s_ = 0.342, P<0.001; Fig. 3). This correlation was stronger for aparasitemic compared to parasitemic individuals (r_s_ = 0.375, P<0.001 and r_s_ = 0.119, P = 0.153, respectively). No correlation was seen between 3D7 parasite growth and IgG to schizont extract (r_s_ = −0.01, P = 0.887). Associations between parasite growth and IgG to MSP1-42 were similar to those seen for responses to schizont extract. A positive correlation between W2mef growth (i.e. reduced growth inhibition) and IgG to MSP1-42 was observed in both aparasitemic and parasitemic individuals (r_s_ = 0.301, P = 0.005 and r_s_ = 0.265, P = 0.056, respectively). No correlation existed between 3D7 growth and IgG to MSP1-42 among samples (r_s_ = −0.055, P = 0.527). Additionally, no correlation was found between antibodies to recombinant MSP1-19 and inhibition of 3D7 growth (data not shown). Similar results were obtained when the analysis was restricted to samples from children (data not shown); there was a weak positive correlation between antibodies to schizont extract or MSP1-42 and parasite growth using W2mef, but not 3D7.

Mean levels of inhibition were not significantly different between parasitemic versus aparasitemic individuals. In the 6 months following sample collection, 41 individuals (40 children, 1 adult) had at least one episode of symptomatic malaria, and survival analysis was performed for children only (2–5 years, n = 43 and 6–14 years, n = 57). As expected, older children had a reduced risk of symptomatic malaria compared with younger children (HR = 0.24, P<0.001). There was no significant association between inhibition of either 3D7 or W2mef growth by samples and reduced risk of subsequent malaria with or without adjustment for age.

**Figure 4:**
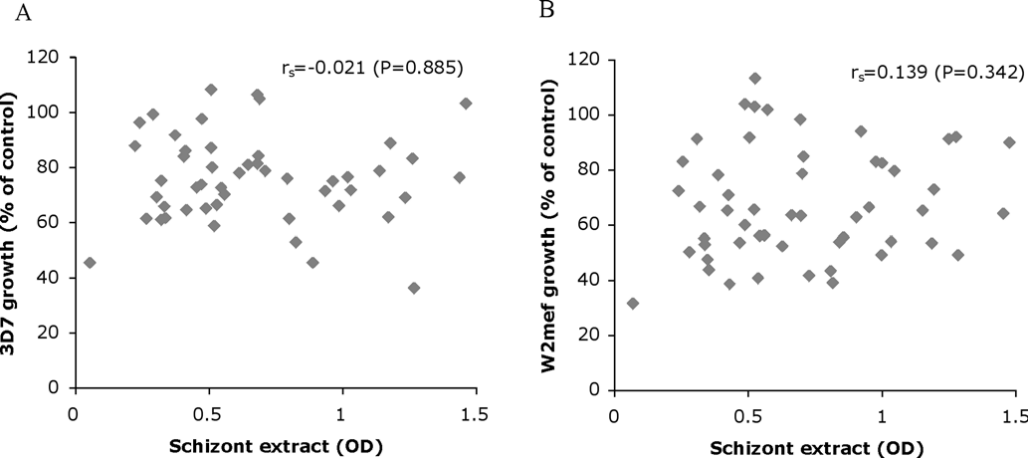
Correlation between IgG to schizont protein extract measured by ELISA and *in vitro* growth inhibition using purified immunoglobulins. A 3D7 parasite line. B W2mef parasite line. Samples used in the assays were immunoglobulins purified using ammonium sulphate precipitation from Ngerenya 1998 serum samples (n = 52). r_s_ represents Spearman's rank correlation coefficients.

The contrasting associations with age and exposure between growth-inhibitory antibodies and total antimalarial antibodies led us to further examine methods used to measure inhibitory antibodies and to validate our findings. Immunoglobulins were purified from an additional 52 sera samples of the Ngerenya 1998 cohort, chosen to achieve a group of children (≤12 years, n = 30) for comparison with adults (>12 years, n = 22), and tested for growth inhibition in single and two-cycle growth-inhibition assays. Again, we found little correlation between 3D7 or W2mef growth and IgG to schizont extract (Fig. 4. A, B); there was a positive correlation between W2mef growth and IgG to schizont extract for aparasitemic individuals (r_s_ = 0.67, n = 9; P = 0.047). Inhibition of growth was not significantly different for adults compared to children (Eg. mean W2mef growth±SD; adults = 70.7±21.8% versus children = 65.6±18.7, P = 0.365) or for high versus low responders to schizont extract (71.3±19.3% versus 66.6±21.1%, respectively, P = 0.461). We typically performed GIAs over two cycles of parasite replication because we have found this increases the sensitivity of the assay [38]. We found a strong correlation between one and two-cycle assays run in parallel for both W2mef and 3D7. The level of growth inhibition was greater for two-cycle compared to one-cycle assays. When samples were tested in single-cycle assays, there were no significant associations between inhibition results and age, parasitemia or ELISA data. Additionally, a subset of 80 samples from the Ngerenya 1998 cohort were independently dialysed and tested in GIAs against 3D7 and W2mef. Similar to our earlier results, little association with age was observed and mean inhibition of W2mef was greater than for 3D7.

### Breadth and strength of growth inhibition responses according to age and exposure

To examine the breadth of response against both parasite lines as well as the strength of growth inhibition among samples from Ngerenya 1998 cohort we defined individuals as ‘strong inhibitors’ (n = 45), ‘intermediate inhibitors’ (n = 40) and ‘weak inhibitors’ (n = 51) based on their activity against both parasite lines. The proportion of strong inhibitors decreased with age (P = 0.009)(Table 1). In addition, the median [inter-quartile range, IQR] IgG level to schizont extract was significantly lower for strong inhibitors (0.19 [0.1–0.51]) compared to intermediate (0.19 [0.01–0.53]) or weak inhibitors (0.36 [0.21–0.78], P = 0.037). There was no evidence of association between breadth and strength of a response with IgG towards MSP1-42 (P = 0.178).

### Profile of inhibitory antibodies in young children with less malaria exposure

To evaluate the influence of differing levels of malaria transmission on the acquisition of inhibitory antibodies, we obtained 237 samples that were collected in a cross-sectional survey in the same community in October 2002 from children aged 1–8 years. The level of malarial transmission in the region was lower in 2002 compared to 1998 [40], which is reflected in the lower proportion of parasitemic individuals at the time of sample collection (Ngerenya 2002, 6.9% versus Ngerenya 1998, 39.3%). To compare growth inhibition between the two studies, analysis was restricted to children of the same age in each study (i.e. 2–8 years; median age of 5.1 years (Ngerenya 2002, n = 207) and 5 years (Ngerenya 1998, n = 61)). Growth inhibition of 3D7 was significantly less in Ngerenya 2002 than Ngerenya 1998 (median [IQR] parasite growth as a percent of control was 100.6 [93.4–105.3] for Ngerenya 2002 versus 43.2 [37.1–48.6] for Ngerenya 1998 samples; P<0.001). For all experiments, the level of growth inhibition by cohort sera was significantly greater than that seen for non-exposed control sera (P<0.001).

Because most Ngerenya 2002 samples were non-inhibitory against 3D7, only an “inhibitory subset” comprised of the top 20% (n = 46) of inhibitory samples (growth relative to controls was <92%) was also tested against W2mef. Median W2mef growth in this inhibitory subset of Ngerenya 2002 child samples was higher (equivalent to reduced inhibition) than that observed for all samples from children (aged 2–8 years) of the Ngerenya 1998 cohort (64.2 [55.8–72.4] versus 32.8 [27.9–36.6] respectively; P<0.001). In considering only the 2002 samples, the inhibitory subset of Ngerenya 2002 samples had higher IgG to schizont extract compared with the remaining less-inhibitory (3D7 growth ≥92%, n = 191) Ngerenya 2002 samples (median [IQR] IgG: 0.16 [0.09–0.57], versus 0.13 [0.04–0.42], respectively, P = 0.038). However, there was no difference in age distribution between the most inhibitory and less-inhibitory Ngerenya 2002 samples (P = 0.599). This suggests that exposure to blood-stage malaria, rather than age per se, is the major determinant for the acquisition of inhibitory antibodies in this cohort of children.

## Discussion

Our findings demonstrate that children exposed to *P. falciparum* can acquire growth-inhibitory antibodies at a young age after little exposure to malaria. It appears that once these antibodies have been acquired they are not boosted by subsequent exposure in the classical manner described for antibodies to blood-stage antigens. In contrast to the age-associated increase in antibodies to schizont extract and MSP1-42, parasite growth-inhibitory antibodies tended to remain stable or decrease with age, despite on-going exposure, and there was no substantial association with active infection. Antibodies to schizont protein extract probably reflect cumulative exposure to malaria and the overall level of blood-stage immunity. Similar to age associations, there was a trend towards declining growth-inhibitory activity as reactivity to blood-stage antigens in the schizont protein extract increased, but the association was not strong. It is important to note that inhibitory antibodies were clearly related to malaria exposure as we found consistently greater inhibition by samples from malaria-exposed individuals compared to non-exposed controls, and inhibition was greater among samples from children collected during a period of higher malaria transmission. Examination of children in the Ngerenya 2002 cohort with lower levels of malaria exposure compared to those children in the Ngerenya 1998 cohort suggested that the acquisition of inhibitory antibodies was ongoing at that time. In the 2002 cohort there was a positive association between antibodies to schizont extract and inhibitory activity and it appeared that inhibitory activity in this group of children had not yet become saturated. Findings among this cohort also confirmed that inhibitory activity can be acquired at an early age and does not require repeated exposure over several years.

The role of growth-inhibitory antibodies against blood-stage *P. falciparum* in immunity to malaria, and their relevance to the progression of disease *in vivo*, remains unclear. In our cohort, growth-inhibitory antibodies were not associated with reduced risk of symptomatic malaria. As expected, age was significantly associated with reduced risk of malaria, yet inhibitory activity was not positively associated with age. The acquisition of inhibitory antibodies at an early age and the relatively stable levels of inhibitory antibodies through childhood suggest that a potential role for these responses in protection from early childhood malaria should be considered. Immunity to severe malaria is acquired relatively quickly after little exposure whereas effective immunity to mild symptomatic malaria is acquired significantly later [2]. It is possible that early immune responses, such as the acquisition of growth-inhibitory antibodies, are important for immunity to severe malaria. Further studies are needed to test this hypothesis.

These findings have important implications for development and evaluation of candidate blood-stage vaccines. It is assumed that antibodies to merozoite antigens should induce inhibitory antibodies, but the relevance of this response to immunity in humans remains to be established. Vaccine trials with merozoite antigens are usually first done among older children with prevention of symptomatic malaria as the end-point; this may not be an appropriate strategy to evaluate these responses. Our results suggest that individual inhibitory antibody responses do not continue to increase in effect once they are acquired, which occurs following limited exposure. This may explain recent findings that immunization of malaria-exposed adults with AMA1 boosted total IgG to recombinant AMA1, but did not increase inhibitory activity [41]. An earlier study in The Gambia similarly found that inhibitory antibodies tended to be lower among adults than children [42], and recent studies by Dent *et al.* in western Kenya report maximum inhibitory activity among young children, which subsequently declines with increasing age [43]. Two prior studies also found no association between growth inhibition by dialysed serum [42] or whole serum [25] and risk of mild malaria episodes, as we found here. On the other hand, some data suggests growth-inhibitory antibodies may contribute to a reduced risk of reinfection. In western Kenya, MSP1-19 specific inhibitory activity of whole serum and total growth-inhibitory activity of dialysed serum was associated with reduced risk of reparasitisation after treatment [23], [43]. Further studies are needed to focus on possible associations between inhibitory antibodies and protection from symptomatic malaria in early childhood.

The use of untreated serum/plasma by some studies may have affected the measurement of inhibitory antibodies and the associations with clinical parameters. Non-specific growth-inhibitory factors, such as antimalarials, have been reported in untreated samples [38], [44]; therefore we dialysis-treated all samples prior to use in our study. Comparable inhibitory effects of dialysed serum versus immunoglobulin isolated from serum have been shown previously [38]. In the present study, similar associations between growth-inhibitory activity of samples and other parameters were found using dialysed serum or purified immunoglobulins suggesting that antibody rather than other serum factors is the major mediator of growth inhibition in our assays. Chloroquine was the drug most likely to have been used for treatment of malaria in the population at that time [45] and we confirmed that chloroquine was removed by serum dialysis (data not shown). Furthermore, we found that W2mef was resistant to common anti-malarial drugs at concentrations that could be expected in serum following treatment for malaria (McCallum, Richards, Wilson, unpublished data). Therefore the presence of antimalarials in samples is unlikely to be a factor influencing our growth inhibition results.

In our studies, associations between age, exposure, and inhibitory antibodies were established by extensive testing of two different parasite lines. Additionally, no association between age and level of growth inhibitory activity was found using W2mef variants with different invasion phenotypes (Persson, McCallum, Beeson, unpublished data). Interestingly, W2mef was consistently inhibited to a greater degree than 3D7. Differences in the inhibitory activity against different parasite lines may be explained by differences in the level of exposure in the population to key epitopes expressed by these isolates. 3D7 and W2mef are known to differ in allelic variants of several important antigens, such as AMA1, and use different ligands for erythrocyte invasion [12], [46]. Recent studies suggest that variation in the use of erythrocyte invasion pathways may act as a mechanism that facilitates immune evasion [13].

Differences in the acquisition and maintenance of ELISA-measured antibodies compared with growth-inhibitory antibodies may be explained by several factors. Human antibody responses to recombinant merozoite antigens have been reported to be short lived among children [47], [48], but appear to stabilise in older children and adults; this response needs to be investigated with respect to inhibitory antibodies. Maintenance of inhibitory antibodies at a sufficient concentration for functional activity may require exposure to a threshold parasite density that is reached in young children during infection, but not in adults and older children who more effectively control and suppress parasite density. A further consideration is that the polyclonal antibody response to merozoite antigens includes inhibitory and non-inhibitory antibodies; repeated exposure may lead to non-inhibitory antibodies reaching a greater concentration or higher affinity than inhibitory antibodies, especially if inhibitory epitopes are fewer in number than non-inhibitory epitopes. Others have reported the acquisition of antibodies that can block the activity of inhibitory antibodies [28], which may impede the development of inhibitory activity with increased exposure; presently, this effect is not well understood. Antibodies to merozoite antigens may also act by antibody-dependent cellular inhibition of parasite growth rather than by directly inhibiting invasion [49].

The lack of association between high levels of IgG to recombinant MSP1-42 or MSP1-19 and growth-inhibitory activity raises concerns regarding the accurate measurement of naturally-acquired and vaccine-induced immunity. The sum effects of multiple antibodies towards different merozoite antigens in serum samples may confound associations between measurements of growth inhibition and antibodies to a single antigen. However, in our study children with high ELISA antibodies to MSP1-42, or MSP1-19, often lacked inhibitory activity, suggesting that ELISA is a not a reliable measure of inhibitory activity. Vaccination of monkeys with MSP1-42 induced growth-inhibitory antibodies and protected against *P. falciparum* challenge [33]. However, vaccination of human volunteers with recombinant MSP1-42 induced inhibitory antibodies only in a subset of individuals, despite high rates of seroconversion to the recombinant antigen [31], [32]. Further studies are clearly needed to understand the relationship between antibody reactivity in standard immunoassays and growth-inhibitory activity.

These findings have important implications for understanding and measuring immunity, and for the development and evaluation of blood-stage vaccines. Our results suggest that the acquisition of functional antibodies differs from that which has been predicted from studies of acquired immunity using standard immunoassays. Antibodies can be acquired at an early age after limited exposure to malaria, but do not demonstrate continued boosting or enhancement with ongoing exposure. Furthermore, antibodies measured by ELISA to potential targets of inhibitory antibodies, such as MSP1-42 or MSP1-19, showed little correlation with growth-inhibitory activity. Results from this work suggest that future investigations on inhibitory antibodies are needed among younger children at the time when they are first experiencing malaria episodes.
